# Porous High-Entropy Oxide Anode Materials for Li-Ion Batteries: Preparation, Characterization, and Applications

**DOI:** 10.3390/ma17071542

**Published:** 2024-03-28

**Authors:** Lishan Dong, Yihe Tian, Chang Luo, Weimin Zhao, Chunling Qin, Zhifeng Wang

**Affiliations:** “The Belt and Road Initiative” Advanced Materials International Joint Research Center of Hebei Province, School of Materials Science and Engineering, Hebei University of Technology, Tianjin 300401, China; 201811801015@stu.hebut.edu.cn (L.D.); 202331802070@stu.hebut.edu.cn (Y.T.); cluohebut@126.com (C.L.); clqin@hebut.edu.cn (C.Q.)

**Keywords:** high-entropy oxide, porous material, Li-ion battery, anode

## Abstract

High-entropy oxides (HEOs), as a new type of single-phase solid solution with a multi-component design, have shown great potential when they are used as anodes in lithium-ion batteries due to four kinds of effects (thermodynamic high-entropy effect, the structural lattice distortion effect, the kinetic slow diffusion effect, and the electrochemical “cocktail effect”), leading to excellent cycling stability. Although the number of articles on the study of HEO materials has increased significantly, the latest research progress in porous HEO materials in the lithium-ion battery field has not been systematically summarized. This review outlines the progress made in recent years in the design, synthesis, and characterization of porous HEOs and focuses on phase transitions during the cycling process, the role of individual elements, and the lithium storage mechanisms disclosed through some advanced characterization techniques. Finally, the future outlook of HEOs in the energy storage field is presented, providing some guidance for researchers to further improve the design of porous HEOs.

## 1. Introduction

In recent decades, the rapid consumption of fossil fuels has not only led to the depletion of non-renewable resources, but has also caused serious environmental problems, in particular global warming due to greenhouse gas emissions [1]. Although many green and renewable energy sources have been developed, such as wind, water, and solar, most renewable energy sources suffer from discontinuities and regional constraints [2,3]. Therefore, the development of high-performance energy storage devices has become a hot research topic in the new era [4,5,6,7], in which the lithium-ion battery (LIB) is one of the most promising energy storage devices due to the advantages of high energy density, strong cyclic stability, and environmental friendliness.

**Figure 1:**
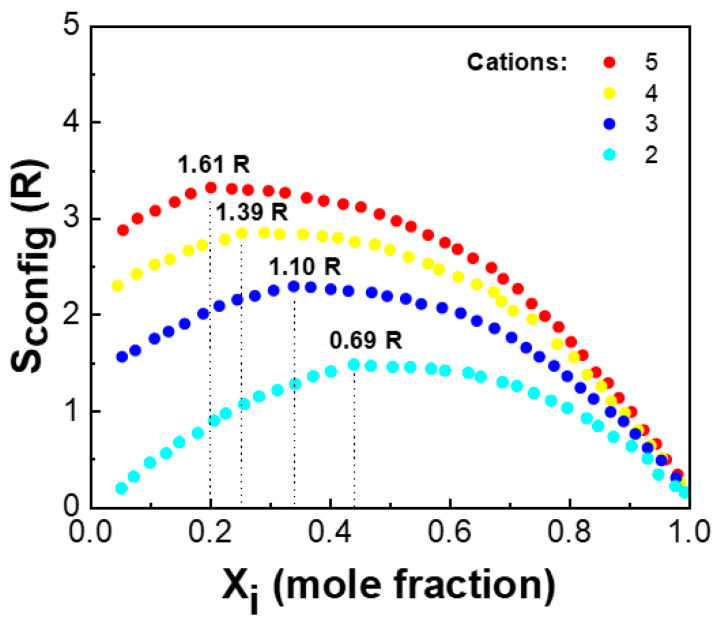
Configurational entropy versus the number of elements [15].

For lithium-ion batteries, the anode plays an important role in improving the cycling stability and electrochemical performance. Commercial graphite no longer meets the demand for high-performance lithium-ion batteries due to its relatively low theoretical capacity (372 mAh g^−1^). In exploring alternative materials for anodes, various types of anode materials, including intercalation-type materials (graphene, Ti_2_O, Li_4_Ti_5_O_12_, etc.), alloy-type materials (Si, Ge, Sn, etc.) [8,9], and conversion-type materials (M_a_X_b_, M = Fe, Co, Cu, Ni, X = O, S, P, N, etc.), have been developed [10]. High-entropy oxides (HEOs) stand out among many high-performance anodes due to their unique entropy-stabilizing effect and multi-component synergy. HEOs are defined as single-phase oxides composed of five or more metallic elements in a 5–35% molar ratio [11,12,13]. The increase in the number of constituent elements leads to an increase in the configurational entropy (*S_config_*), which is one of the discriminators of HEOs compared to other materials. The magnitude of the conformational entropy (*S_config_*) can be quantified by the following equation [14]:

(1) $$S_{config}=-R\left[{\left(\sum_{i=1}^{N}x_{i}lnx_{i}\right)}_{cation-site}+{\left(\sum_{j=1}^{N}x_{j}lnx_{j}\right)}_{anion-site}\right]$$

(2) $$S_{\mathrm{co}nfig}=R\ln N$$

where R represents the molar gas constant (8.314 J/mol·K), N represents the number of constituent elements, $x_{i}$ represents the cation molar fraction, and $x_{j}$ represents the anion molar fraction. Compared to the cation site, since only one anion (oxygen ion) exists in HEOs, the effect of the anion on the configurational entropy is negligible, and the configurational entropy of the solid solution reaches a maximum when the constituent elements are in equimolar ratios, as shown in Equation (2). Figure 1 demonstrates the conformational entropy of the system versus the number of group elements and the molar ratio of the group elements [15]. The maximum values of the conformational entropy of the material, when the number of constituent elements is 2, 3, 4, and 5, are 0.69, 1.10, 1.39, and 1.61 R, respectively.

Usually, the configurational entropy must be greater than 1.5 R in order to construct an entropy-stabilized oxide. Based on the magnitude of the configurational entropy, Murthy et al. [16] defined *S_config_* ≥ 1.5 R as high entropy, 1 R ≤ *S_config_* ≤ 1.5 R as medium entropy, and *S_config_* < 1 R as low entropy. However, *S_config_* ≥ 1.5 R cannot be the only parameter for the design of high-entropy oxides. For example, (Zr_0.2_Ce_0.2_Hf_0.2_Sn_0.2_Ti_0.2_)O_2_ [17] and (La_0.2_Gd_0.2_Nb_0.2_Sm_0.2_Y_0.2_)MnO_3_ [18] underwent a reversible transition from a single-phase structure to a multiphase mixture when reheated at relatively low temperatures, indicating that TΔS_mix_ was still insufficient to withstand enthalpy-driven phase separation when *S_config_* ≥ 1.5 R. The above categorization, therefore, is only applicable at high temperatures (>800 °C) and not at low temperatures, further illustrating the importance of the temperature (T) for high-entropy oxide materials with a single-phase structure.

In 2015, Rost et al. [19] succeeded in synthesizing quinary (Mg,Ni,Co,Cu,Zn)O systems with different cationic compositions for the first time, and the single-phase oxide systems were prepared by using entropy-driven structural stabilization effects in terms of iso-atomic ratios for the compositional design. In 2016, Beradan et al. [20] coined a more general term, “high-entropy oxides”, and reported two HEOs, (Mg,Co,Ni,Cu,Zn)_1−_Li_x_O and (Mg,Co,Ni,Cu,Zn)_1−x_Na_x_O. By testing the electrochemical properties of these two oxides, it was found that they have ultra-high electronic conductivity at room temperature. After that, HEOs were developed rapidly in the field of electrochemical energy storage, attracting extensive attention from a large number of scholars. 

In addition to research papers, reviews focusing on HEOs have gradually increased in recent years [11,12,13,14,21]. Liu et al. [12] reported the application of HEOs in the field of lithium-ion battery anodes, which introduced individual element functions and component design, electrochemical mechanism, advanced synthesis, and modification techniques, etc. However, the review did not focus on porous HEOs, nor on the preparation methods and structural characteristics of such materials. Lin et al. [14] reported the progress made in the field of high-entropy ceramics for reversible energy storage, including the synthesis, processing routes, and electrochemical properties of anode and cathode materials. Likewise, the HEOs the review focused on were not porous materials. Liu et al. [21] reported the application of transition metal oxides and carbon materials with a three-dimensionally ordered porous structure in energy storage, and highlighted the effect of a porous structure on the electrochemical properties of electrode materials. However, the material the review focused on was not HEOs. In addition, the reviews did not focus on the role of advanced in situ characterization techniques in revealing the crystal structure stability/transformation of HEOs. Therefore, an overview of the above aspects is very necessary. In this review, we present in detail the latest discoveries of porous HEOs in recent years and their achievements in the field of lithium-ion battery anodes. The applications of some advanced testing techniques, such as in situ transmission electron microscopy (TEM), in situ X-ray absorption spectroscopy (XAS), and electron energy-loss spectroscopy (EELS), on HEOs are disclosed. A detailed review on the phase transition mechanism of HEO anodes during cycling is also presented. These aspects make this review have a special point of view not provided by previous reviews related to HEOs, allowing this review to be able to provide some reference to scholars working in the porous material, HEO, and LIB fields.

## 2. Preparation Methods of Porous HEOs

**Figure 2:**
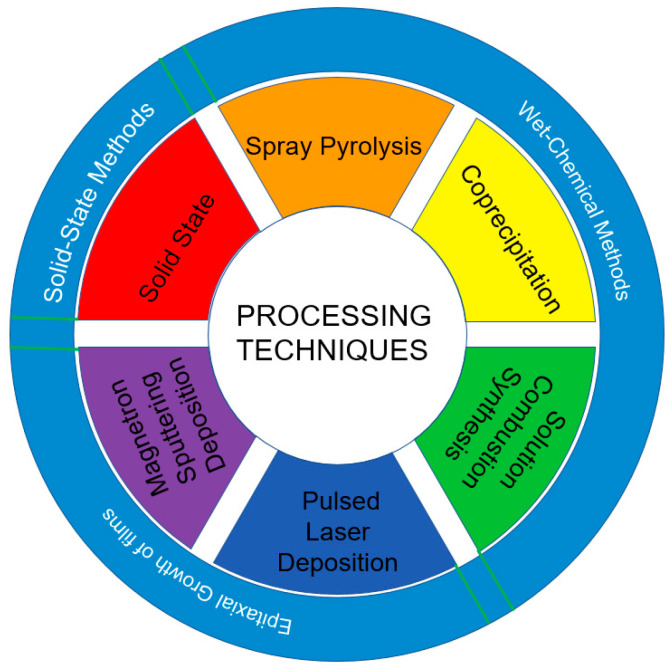
Schematic diagram of the process of preparing high-entropy oxides.

Currently, the preparation techniques of high-entropy oxides can be categorized according to the initial feedstock as well as the application. As shown in Figure 2, the preparation methods of HEOs include solid-state reaction method (high-temperature solid state reaction) [22,23], wet chemical methods (spray pyrolysis, co-precipitation, and solution combustion synthesis) [24,25,26,27,28,29,30], epitaxial growth of films (pulsed laser deposition and magnetron sputtering deposition) [31,32], and so on. This section focuses on the specific flow and characteristics of the process for the preparation of porous HEOs. 

### 2.1. Solid-State Reaction Method

The solid-state reaction method (SRM) is one of the most common processes for the preparation of porous HEOs, in which five or more prepared oxides are mixed and then calcined after thorough mixing using a ball mill or grind [22]. Mechanical activation by ball milling and grinding is of great importance for the synthesis of HEOs, not only to reduce the size of the mixtures but also to facilitate the rapid diffusion of atoms [23]. After mechanical activation, the collected powders are subjected to high-temperature calcination to induce the formation of single-phase HEOs by increasing the temperature to overcome the enthalpy of mixing. According to the relevant literature [33], the critical temperature for the formation of single-phase HEOs is around 800 °C. In addition to the calcination temperature, the calcination time is also an important influencing factor. At high temperatures, too long a calcination time can lead to particle aggregation and phase transform. Conventional calcination processes in air tend to require a longer heating time, and as a result, the synthesized HEOs show particle accumulation with a typical porous structure [34]. For instance, Chen et al. [35] reported the preparation of a new spinel (Mg_0.2_Ti_0.2_Zn_0.2_Cu_0.2_Fe_0.2_)_3_O_4_ HEO by the solid-state reaction method, which generated oxide nanoparticles with a specific surface area of 12.31 m^2^ g^−1^ and pore sizes in the range of 3–20 nm. Wang et al. [22] prepared a spinel-structured (FeCoCrNiMn)_3_O_4_ HEO by the solid-state reaction method. The preparation process requires calcination at high temperatures (>800 °C) for 12 h to produce single-phase structure, resulting in the significant agglomeration of particles. However, spark plasma sintering and reactive flash sintering allow for rapid heating and a shorter heating time, which are more conducive to the formation of dense HEOs. Although the solid-state reaction method presents a simpler way to prepare HEOs, it often requires longer heating times to ensure the homogeneous mixing of elements, leading to undesired phase segregation and the excessive growth of particles, which disrupts precise size and morphology control. 

### 2.2. Wet Chemical Methods

Wet chemical methods are methods for preparing high-entropy oxides using metal salts as precursors. Compared to the solid-state reaction method, the metal salt precursor is a homogeneous solution with mixed elements at the atomic level of entropy; so, a relatively low temperature of calcination is required to prepare single-phase HEOs. Nebulized spray pyrolysis (NSP) is an aerosol process that has been used to prepare high-entropy oxides with a highly crystalline structure [36]. The metal salt solution is transported as a mist spray through a carrier gas (oxygen) to the heating zone in the tube furnace, where the precursor is converted to the desired crystalline oxide under the action of high temperatures. Flame spray pyrolysis (FSP) requires a higher concentration of the precursor solution compared to spray pyrolysis (1 mol/L vs. 0.1 mol/L) due to its lower production rate as well as higher yield [37]. Sarkar et al. [38] prepared (Co_0.2_Cu_0.2_Mg_0.2_Ni_0.2_Zn_0.2_)O with a rock salt structure by NSP, confirming that NSP can be used to prepare HEOs with high crystallization. 

Solution combustion synthesis (SCS) is a special method of preparing HEOs by an exothermic reaction between metal nitrate and fuel. The method does not have a subsequent calcination process and, therefore, offers a low energy consumption as well as excellent nanostructure control. In addition, a large number of gaseous by-products are formed, resulting in the formation of porous HEOs [39,40]. He et al. [41] prepared nano-porous (Fe_0.2_Co_0.2_Ni_0.2_Cr_0.2_Mn_0.2_)_3_O_4_ HEOs by the low-temperature solution combustion method. Benefiting from the nano-porous structure and high grain boundary density, (Fe_0.2_Co_0.2_Ni_0.2_Cr_0.2_Mn_0.2_)_3_O_4_ exhibits superior stability.

The co-precipitation (CP) method has gained importance in the preparation of HEOs due to homogeneous mixing at the atomic scale and the excellent tunability of the particle morphology of the prepared powders [42]. Biesuz et al. [43] prepared single-phase (Mg,Co,Ni,Cu,Zn)O high-entropy oxides by the CP method. The method is based on the use of aqueous nitrate solutions of five metals, with NaOH as a precipitant, to induce the precipitation of the sample. It is then calcined at 500 °C for 1 h to decompose the precipitant and hydrated oxides. It is noteworthy that a highly alkaline environment is required for the complete precipitation of Mg. The high-entropy oxides prepared by the CP process have a dense microstructure and possess a uniform distribution of small voids with a relative density close to 97%.

The porous HEOs prepared by the wet chemical method have a stronger crystallization, and this method is more suitable for the preparation of porous HEOs due to the gas produced in this reaction process, in which the porous structure can effectively improve the electrochemical performance of HEOs. The limitation of this method is that, when scaling up production, the uniformity of the product may be affected. In addition, the residual salt composition may reduce the performance of the material.

### 2.3. Epitaxial Growth of Films

The characteristic of this method is that the thickness of the prepared HEOs is relatively thin, and it needs to be combined with etching technology to form a porous structure. Pulsed laser precipitation (PLD) involves a physical vapor-phase precipitation process. High-entropy oxides prepared by solid-state reactions and wet chemistry as described above are used as targets, and HEO thin films of varying thicknesses are grown on single-crystal substrates using pulsed light precipitation [31,44]. Sharma et al. [45] reported a study on the preparation of chalcogenide Ba(Zr_0.2_Sn_0.2_Ti_0.2_Hf_0.2_Nb_0.2_)O_3_ HEO single-crystal films by the PLD process. The specific method is to prepare HEOs as targets by a conventional high-temperature solid-state reaction, and then ablate a small amount of high-entropy oxides using high-energy pulsed laser focusing on the high-entropy oxides on the SrTiO_3_ and MgO single-crystal substrates. Atomic vapor deposition is utilized to form a thin film on the heated substrate. Unlike PLD, magnetron sputter deposition (MSD) can be used to prepare ultrafine nanocrystalline HEO films by the sputter growth of thin films. Yang et al. [32] prepared (Al_0.31_Cr_0.2_Fe_0.14_Ni_0.35_)O HEO thin films using the magnetron sputtering precipitation method and found that HEO thin films contained different content quantities of He. The He injection caused the grain boundary cavities to have a significant effect on the mechanical properties of the HEOs.

**Figure 3:**
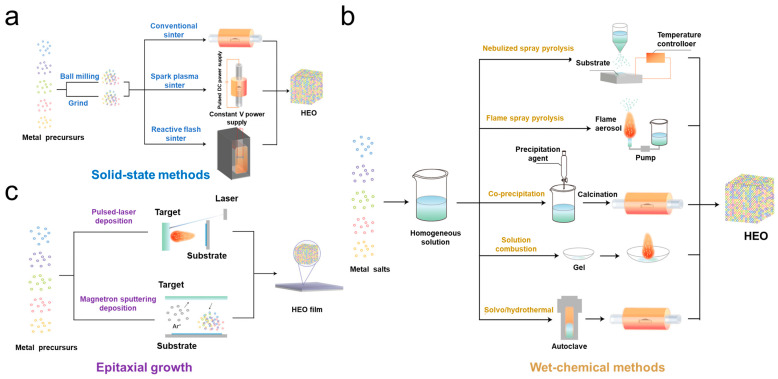
Synthesis process of high-entropy oxides: (**a**) solid-state methods; (**b**) wet chemical methods; and (**c**) epitaxial growth [46].

Lee et al. [46] presented a schematic diagram of three types of processes for the preparation of HEOs, as shown in Figure 3. Although the existing processes for the preparation of high-entropy oxides are established, there is still much room for improvement. In order to achieve the synthesis of high-quality HEOs, the following requirements should be met: (1) the precursors need to be loosely stacked to avoid densification; (2) the calcination temperature as well as the time need to be accurately controlled in terms of the particle size; and (3) low energy consumption and rapid synthesis. 

## 3. Characterization of Porous HEOs

### 3.1. Crystal Structures of Porous HEOs

**Figure 4:**
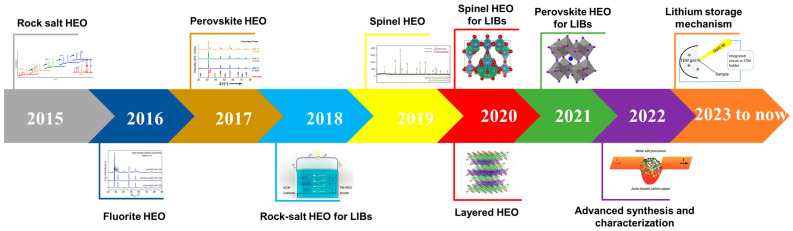
Timeline of the explored HEO structures and applications in Li-ion battery anodes [12,15,19,34,48,57,58,59,60,61].

Since the first high-entropy oxide was developed in 2015 [19], HEOs with different crystal structures have been developed, including rock salt [26,47], spinel [48], perovskite [49], fluorite [50,51], and layered O3-type [52,53]. There is a great variety of crystal structures and there are the thousands of ways of combining the elements that enrich the field of application of HEOs (thermoelectric applications, electrochemical energy storage and catalysis, etc.). In the field of lithium-ion battery anodes, the first one to be studied was a HEO with a rock salt structure, which attracted wide attention from researchers due to its unique “entropy stability effect” and ultra-high electronic conductivity [38]. In addition, spinel-structured HEOs have a higher theoretical capacity when used as an anode in lithium-ion batteries due to the inclusion of two Wyckoff sites, allowing for more valence changes in the metal cation. In particular, the emergence of porous spinel high-entropy oxides with a unique porous structure that improves the electrochemical properties of HEOs has attracted increasing attention [54,55,56]. Figure 4 shows a timeline of the development of HEO crystal structures and their application in the field of lithium-ion battery anodes since 2015 [12,15,19,34,48,57,58,59,60,61]. In addition to the above-mentioned crystal structures, various crystal structures of HEOs, such as magnetite-structured Ba(Fe_6_Ti_1.2_Co_0.2_In_1.2_Ga_1.2_Cr_1.2_)O_19_ [62], monoclinic-structured (Yb_0.25_Yi_0.25_Lu_0.25_Er_0.25_)_2_SiO_5_ [63], and rutile-structured (AlCrNbTaTi)O_2_ [64], have been developed, but since they have not been reported in the field of lithium-ion batteries, they are not described in detail in this review. In recent years, various types of in situ techniques have been successively applied to reveal the lithium storage mechanism when HEOs are used as anodes in lithium-ion batteries.

### 3.2. Structural Characterization of Porous HEOs

It is well-known that a specific surface area and pore volume can influence the electrochemical performance of porous HEOs [39]. HEOs tend to exhibit different pore structures after experiencing different preparation processes. Porous HEOs prepared by solid-state reaction methods present mostly a nanoparticle structure; so, their pores are mainly formed by the accumulation of nanoparticles. The pore size is related to the size of the particles [35,65]. In addition, porous HEOs prepared by wet chemical methods tend to exhibit a hierarchical porous structure due to the release of large amounts of gas during the preparation process [66]. In this way, porous HEOs prepared by wet chemical methods often display a higher specific surface area than those prepared by solid-state reaction methods. Table 1 lists the key parameters of the pore structure of porous HEOs by the different preparation methods. In general, an abundant pore structure with a high specific surface area facilitates charge transfer, reduces the ion diffusion path length, and provides enough space to accommodate volume changes during cycling, thus extending the cycle life and enhancing the electrochemical performance of lithium-ion batteries [67]. Yan et al. reported a porous (Co_0.2_Cu_0.2_Mg_0.2_Ni_0.2_Zn_0.2_)O HEO prepared by solution combustion, which displayed macropores along the ligament networks as well as mesopores on the ligaments, with a specific surface area of up to 43.54 m^2^ g^−1^ [68]. Li et al. prepared (FeCuCrMnNi)_3_O_4_ HEOs by spray pyrolysis with a homogeneous hollow porous structure, which presented excellent electrochemical properties [69]. 

### 3.3. Phase Structure Characterization of Porous HEOs

**Figure 5:**
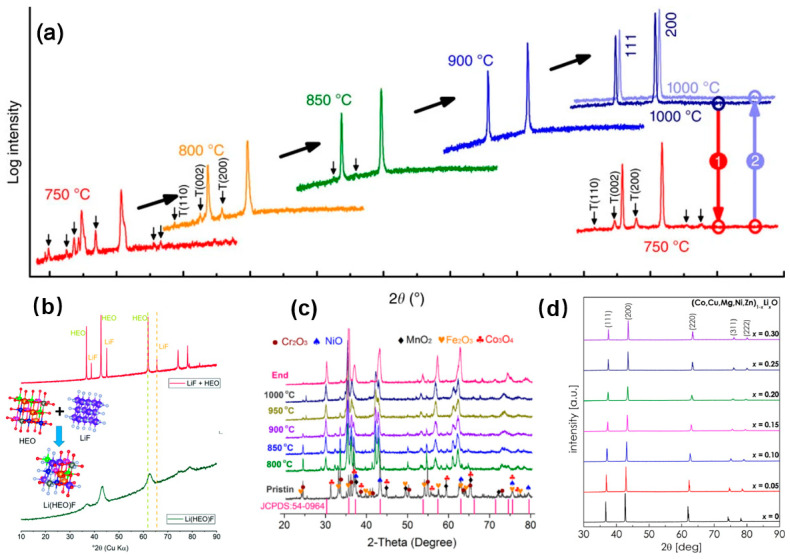
(**a**) XRD diffraction patterns for (Mg,Ni,Co,Cu,Zn)O [19]. (**b**) XRD patterns of both a physical mixture of LiF and (Co_0.2_Cu_0.2_Mg_0.2_Ni_0.2_Zn_0.2_)O, and the as-synthetized Li_x_(Co_0.2_Cu_0.2_Mg_0.2_Ni_0.2_Zn_0.2_)OF_x_ sample [78]. (**c**) XRD patterns of the pristine oxide precursor and insitu HTXRD patterns of (FeCoNiCrMn)_3_O_4_ (within the range of 800–1000 °C) [22]. (**d**) XRD patterns of the (Co,Cu,Mg,Ni,Zn)_1−x_Li_x_O HEOs in the selected range [79].

An important criterion for verifying the synthesis of HEOs is the single-phase structure, which can be confirmed by X-ray diffraction (XRD) analysis [75,76,77]. As shown in Figure 5a, Rost et al. [19] used XRD to test the transformation process in the phase composition of the sample (a mixture of five kinds of oxides with equal atomic ratios) in an air ambient environment up to 1000 °C, during their first synthesis of (Mg,Ni,Co,Cu,Zn)O HEOs. At a relatively low temperature (750 °C), the sample showed a multiphase structure, while characteristic peaks of the single-phase rock saltpeter structure began to appear as the temperature increased. When the temperature reached 850 °C, the sample was completely transformed into a single-phase rock salt structure, and the crystallinity became higher with the further increase in the temperature. It was also found that, when the temperature was reduced from 1000 °C to 750 °C and then increased to 1000 °C, a reversible transition between a single phase and multiphase was observed, proving that the conformational entropy is the main driving force for this reversible phase transition. Figure 5b shows the XRD patterns of LiF and (Co_0.2_Cu_0.2_Mg_0.2_Ni_0.2_Zn_0.2_)O HEOs to prepare a novel high-entropy material of Li_x_(Co_0.2_Cu_0.2_Mg_0.2_Ni_0.2_Zn_0.2_)OF_x_ containing polyanions and cations simultaneously. The two different phases were then ball-milled for 24 h to form a single-phase rock salt structure, further confirming that conformational entropy can drive the formation of single-phase structures [78]. In addition, high-temperature XRD is often used to probe the optimal temperature for high-entropy oxide synthesis. For example, Wang et al. [22] used high-temperature XRD to test the conditions for the synthesis of a single-phase spinel-structured (FeCoNiCrMn)_3_O_4_ (Figure 5c). XRD analysis can also be used to analyze the effect of different doping contents on the phase and structure of Li-doped transition metal oxides, as reported by Moździerz et al. Figure 5d shows the XRD patterns of (CoCuMgNiZn)_1−x_Li_x_O when x is 0.05, 0.1, 0.15, 0.20, 0.25, and 0.30. It was found that the peaks corresponding to the (311) and (220) crystal faces shifted to higher angles with the increase in the Li content, indicating a tendency for the lattice to shrink, which is related to charge compensation [79].

### 3.4. Mophological Characterization of Porous HEOs

**Figure 6:**
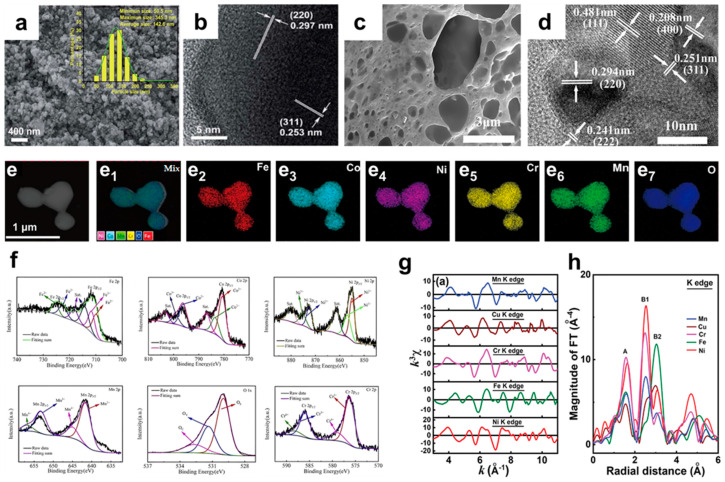
(**a**) SEM image and (**b**) HRTEM image of (Mg_0.2_Ti_0.2_Zn_0.2_Cu_0.2_Fe_0.2_)_3_O_4_ [35]. (**c**) SEM image and (**d**) HRTEM image of (Cr_0.2_Fe_0.2_Co_0.2_Ni_0.2_Zn_0.2_)_3_O_4_ [56]. (**e**) EDS mapping of (FeCoNiCrMn)_3_O_4_ [22]. (**f**) The high-resolution XPS spectrogram of (FeCoNiCrMn)_3_O_4_ [80]. (**g**) K-edge k^3^-weighted EXAFS spectra and (**h**) Fourier transform magnitude spectra of all constituent elements in (CrMnFeNiCu)_3_O_4_ [81].

Physicochemical properties, such as morphology, crystal structure, crystal defects, and surface chemistry, play a crucial role in understanding HEOs. According to the literature, the morphology of HEOs mainly includes particle, film, porous structure, and so on. Chen et al. [35] reported a new spinel-structured (Mg_0.2_Ti_0.2_Zn_0.2_Cu_0.2_Fe_0.2_)_3_O_4_ high-entropy oxide prepared by the solid-state reaction method, whose scanning electron microscopy (SEM) images and high-resolution transmission electron microscopy (HRTEM) images are shown in Figure 6a and b, respectively. The (Mg_0.2_Ti_0.2_Zn_0.2_Cu_0.2_Fe_0.2_)_3_O_4_ HEO is composed of irregular spherical nanoparticles with an average size of 143 nm. The lattices with intervals of 0.297 and 0.253 nm are indexed as the (220) and (311) crystal planes of the spinel structure, confirming that the synthesized HEOs are spinel-structured. Porous HEOs have received wider attention due to their improved specific surface area. Yang et al. [56] successfully prepared spinel (Cr_0.2_Fe_0.2_Co_0.2_Ni_0.2_Zn_0.2_)_3_O_4_ HEOs with a porous structure using the sol-gel method, whose SEM image is shown in Figure 6c. A large amount of gas is released during the heating process, resulting in a porous morphology and foamy agglomerates. The high-resolution TEM image in Figure 6d shows high crystallinity and the tested lattice spacing is consistent with the spinel phase. Wang et al. [22] confirmed the high-entropy state of the synthesized samples by energy dispersive spectroscopy (EDS) mapping, in which all elements in the synthesized (FeCoNiCrMn)_3_O_4_ were uniformly distributed on the nanoparticles (Figure 6e). X-ray photoelectron spectroscopy (XPS) is commonly used to analyze the chemical properties of material surfaces. As shown in Figure 6f, Xiao et al. tested the XPS spectra of (FeCoNiCrMn)_3_O_4_. The results show that spinel high-entropy oxides allow a higher range of valence states to occur relative to the rock salt structure, which increases the number of electrons transported and can improve the specific discharge capacity of HEOs [80]. In addition, all elements in HEOs are randomly distributed in crystals. In order to understand the local coordination environments of the constituent elements of HEOs, Luo et al. [81] tested (CrMnFeNiCu)_3_O_4_ high-entropy oxides using X-ray absorption fine spectroscopy (EXAFS). As shown in Figure 6g and h, the FT spectrum reveals two main signals (A and B) corresponding to these metal–oxygen bonding and metal–metal bonding energies, where signal B has two subpeaks related to the scattering of metal atoms at the octahedral and tetrahedral positions, respectively. The test results show that the spectra of all elements have similar features indicating that all elements are randomly distributed in the spinel crystals, while the elemental spectra of Ni and Cr present a higher signal A, indicating that these two elements are more inclined to occupy the octahedral sites.

## 4. Application of Porous HEOs in Lithium-Ion Battery Anodes

### 4.1. Electrochemical Properties

The growth of the electric vehicle industry poses a vast challenge to lithium-ion batteries. However, commercially available graphite anodes are no longer able to meet the needs of the electric vehicle industry [82,83]. Among the many materials explored for high-performance anodes, transition metal oxides have received considerable attention due to their high theoretical capacity, low cost, and easy preparation, but the vast volume expansion during cycling and their inherent semiconducting nature limit their application [84,85,86]. In this section, we focus on the application of single-phase HEOs as anodes in lithium-ion batteries.

**Figure 7:**
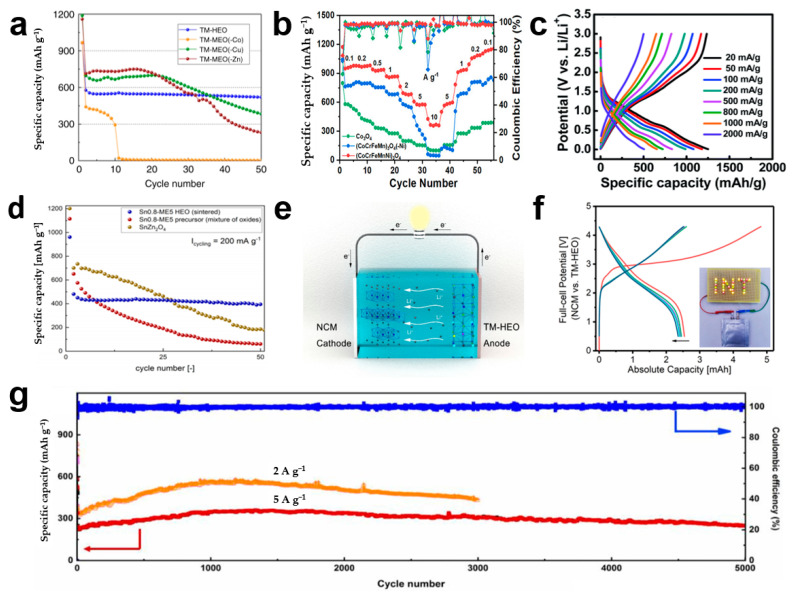
(**a**) Cycling performance of (Co_0.2_Cu_0.2_Mg_0.2_Ni_0.2_Zn_0.2_)O, (Cu_0.25_Mg_0.25_Ni_0.25_Zn_0.25_)O, (Co_0.25_Mg_0.25_Ni_0.25_Zn_0.25_)O, and (Co_0.25_Cu_0.25_Mg_0.25_Ni_0.25_)O at 50 mA g^−1^ [38]. (**b**) Rate capabilities of Co_3_O_4_, (CoCrFeMn)_3_O_4_, and (CoCrFeMnNi)_3_O_4_ [87]. (**c**) Charge/discharge curves of the (CoCrFeMnNi)_3_O_4_ electrode recorded at different rates [88]. (**d**) Cycling performance of Sn_0.8_(Co_0.2_Mg_0.2_Mn_0.2_Ni_0.2_Zn_0.2_)_2.2_O_4_, the HEO precursor, and SnZn_2_O_4_ [89]. (**e**) Diagram of the full cell using an NCM111 cathode and (Cr_0.2_Mn_0.2_Fe_0.2_Co_0.2_Ni_0.2_)_3_O_4_ HEO anode. (**f**) Charge/discharge profiles of a pouch cell in the voltage range of 0.5–4.3 V [57]. (**g**) Cycling performance of (Cr_0.2_Mn_0.2_Fe_0.2_Co_0.2_Ni_0.2_)_3_O_4_ at the current densities of 2 A g^−1^ and 5 A g^−1^ [15].

In 2018, Sarkar et al. [38] fabricated rock salt (Co_0.2_Cu_0.2_Mg_0.2_Ni_0.2_Zn_0.2_)O HEOs for lithium-ion battery anodes and systematically compared the electrochemical performance of five-cation HEOs with four-cation medium-entropy oxides (equal atomic ratios) (Figure 7a). Compared to HEOs, medium-entropy oxides exhibit relatively unstable electrochemical properties, while HEOs exhibit a surprising cycling stability, which is related to the entropy stabilization effect. Furthermore, in order to verify the superior rate performance of HEOs with respect to transition metal oxides, Zhao et al. [87] prepared spinel-structured (Co_0.2_Cr_0.2_Fe_0.2_Mn_0.2_Ni_0.2_)_3_O_4_ HEOs by the solution combustion method and demonstrated that the HEOs have excellent rate properties at different current densities (even at 10 A g^−1^), which is far superior to that of the transition metal oxides Co_3_O_4_ and (CoCrFeMn)_3_O_4_ (Figure 7b). Converted transition metal oxide anodes are known to exhibit significant deterioration in capacitance at high current densities, which is related to the inherently low conductivity and low diffusion kinetics. Nguyen et al. [88] tested the charge/discharge performance of (CoCrFeMnNi)_3_O_4_ at the current density values of 50, 100, 200, 500, 800, and 1000 mA g^−1^, as shown in Figure 7c, with reversible capacities of 1170, 1072, 979, 824, 715, and 649 mAh g^−1^, respectively. Even at 2000 mA g^−1^, it was still able to deliver a 500 mAh g^−1^ discharge capacity. In order to further improve the cycling performance of HEOs, Sn_0.8_(Co_0.2_Mg_0.2_Mn_0.2_Ni_0.2_Zn_0.2_)_2.2_O_4_ conversion-alloy anode electrode materials were prepared by the high-temperature solid phase method by Mozdzierz et al. [89]. As shown in Figure 7d, Sn_0.8_(Co_0.2_Mg_0.2_Mn_0.2_Ni_0.2_Zn_0.2_)_2.2_O_4_ exhibits a surprising cycling stability due to the inclusion of two different lithium storage mechanisms, namely, conversion-type lithium storage and alloy-type lithium storage mechanisms. Wang et al. [57] assembled a coin cell of NC/TM-HEO (Figure 7e) as well as a pouch cell with a similar area loading as that of coin-type batteries to test the potential of HEOs for practical applications. The first five cycles of the charge/discharge performance of a full battery are revealed in Figure 7f, and it can supply power to LED arrays successfully. Figure 7g shows the cycling performance of the (Cr_0.2_Mn_0.2_Fe_0.2_Co_0.2_Ni_0.2_)_3_O_4_ HEO anode, with the highest cycle number of 5000 cycles at the maximum current density of 5 A g^−1^, as stated in the available reports [15].

Porous HEOs show excellent electrochemical performance as anodes for lithium-ion batteries. Table 2 shows the crystal structure, preparation process, and electrochemical performance of different HEOs, which confirms the great potential of high-entropy oxides as anodes for lithium-ion batteries.

### 4.2. Lithium Storage Mechanism

High-entropy oxides have a complex lithium storage mechanism due to their multiple electroactive sites [81,101]. In this section, the latest progress in high-entropy oxide anode is introduced from three aspects, namely, phase transition during cycling process, elemental effects, and the newly proposed lithium storage mechanism.

**Figure 8:**
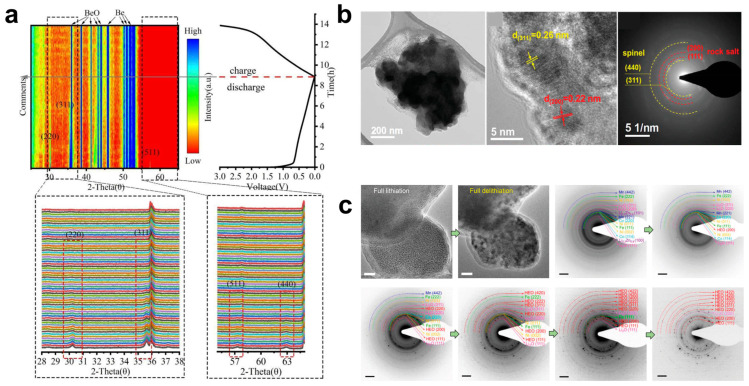
(**a**) In situ XRD results for the first cycle of (FeCoNiCrMn)_3_O_4_ HEO [80]. (**b**) TEM analysis data of (CrNiMnFeCu)_3_O_4_ after 400 charge–discharge cycles [99]. (**c**) TEM snapshots of the evolution of the all-lithium/all-delithiated morphology of (Co_0.2_Ni_0.2_Mn_0.2_Zn_0.2_Fe_0.2_)_3_O_3.2_ and ED patterns used for identifying phase evolution during delithiation [100].

Unlike transition metal oxides, in which the structure collapses after several cycles of the electrode, HEOs have an excellent structural stability. The high-entropy lattice provides a spatial network for the embedded lithium/de-lithium, which ensures the structural stability of HEOs during cycling, resulting in an excellent cycling stability [102,103]. In order to investigate the phase transition of HEO anodes during cycling, various types of in situ as well as ex situ detection techniques have been applied. Among them, in situ XRD, as one of the most common detection techniques, is usually applied to analyze the phase transition during the cycling process. As shown in Figure 8a, Xiao et al. [80] tested the crystal structure transformation of (FeCoNiCrMn)_3_O_4_ during the first charge/discharge process by in situ XRD. Diffraction peaks on the (220), (311), and (511) crystal planes of the spinel structure are found under open circuit voltage conditions and become progressively weaker as the discharge process proceeds. When discharged to 0.01 V, the diffraction peaks of the spinel structure completely disappear and do not reappear during the charging process, probably because the formation of a small number of grains is below the XRD detection ability during the charging/discharging process. However, Zheng et al. [98] found that the diffraction peaks (220), (400), and (440) of the spinel structure were shifted in the peak position during the first discharge by in situ XRD testing of the six-member (FeNiCrMnMgAl)_3_O_4_ HEOs, and the end position of the shift corresponded to the rock salt phase, confirming the coexistence of spinel and rock salt during the charging and discharging processes. As shown in Figure 8b, Patra et al. [99] performed TEM analyses on (CrNiMnFeCu)_3_O_4_ HEOs after 400 charge–discharge cycles. The high-resolution TEM and SAED data confirm the presence of spinel and rock salt phases. In order to be able to better observe the phase transition of HEOs during lithiation/delithiation, Su et al. [100] applied in situ TEM to probe the reaction kinetics and structural evolution of (Co_0.2_Ni_0.2_Mn_0.2_Zn_0.2_Fe_0.2_)_3_O_3.2_ HEOs during cycling processes. As shown in Figure 8c, the characteristic peaks of the rock salt structure of (Co_0.2_Ni_0.2_Mn_0.2_Zn_0.2_Fe_0.2_)_3_O_3.2_ HEOs begins to disappear during lithiation, while the metallic phases of Li_0.2_Zn_0.8_ and Li_2_O tend to appear. Moreover, the rock salt structure phase recovers again after complete delithiation. The above results demonstrate that the key factor for the excellent cyclic stability of the structure is the recovery ability of the structure.

The greatest advantage of HEOs is their customizability, with thousands of element combinations that can be compositionally designed to suit the actual needs [104]. Therefore, understanding the role of each element is of immense value in extending the practical applications of HEOs. In the field of lithium-ion battery anodes, the literature on the role of elements is very sparse, mainly due to the fact that conventional technology does not provide detailed mechanism information for the cycling process. Metal elements in high-entropy oxides are classified into active and inactive elements according to whether or not conversion reactions occur during the charging/discharging processes. For example, the element Mg, mentioned in the first 2018 report on HEOs with rock salt structures for lithium-ion battery anodes, is thought to play a role in stabilizing the rock salt structure due to its inactivity in a specific voltage range without undergoing a conversion reaction [38]. In addition, Chen et al. [105] tested the valence changes of elemental Ti in (Ni_0.2_Co_0.2_Mn_0.2_Fe_0.2_Ti_0.2_)_3_O_4_ before and after cycling by ex situ XPS, and found that there was no change in Ti, thus identifying elemental Ti as inactive. The above results indicate that the addition of inactive elements to high-entropy oxides can play a role in lattice stability.

**Figure 9:**
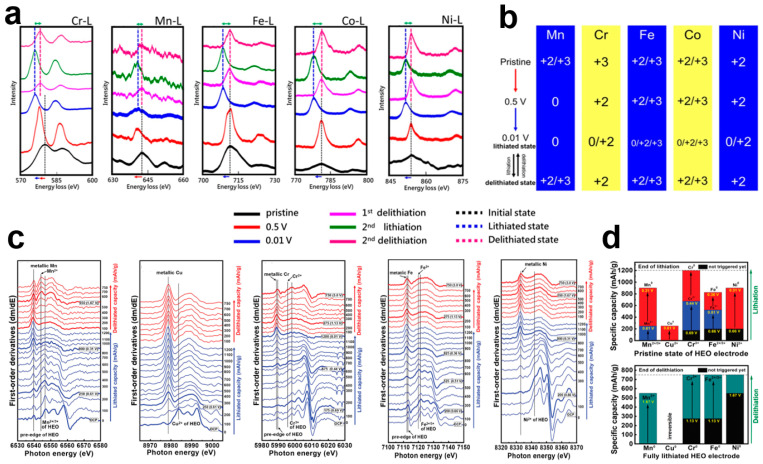
(**a**) EELS analysis results of the (CrMnFeCoNi)_3_O_4_ sample at different charge states and cycle numbers. (**b**) Overview of the changes in valence states for the constituent elements of (CrMnFeCoNi)_3_O_4_ [109]. (**c**) The first-derivative curve of the operando XAS spectra measured during charging/discharging at 150 mA g^−1^ for the (CrMnFeNiCu)_3_O_4_ electrode. (**d**) Summaries describing the transition behavior of all constituent elements in the (CrMnFeNiCu)_3_O_4_ electrode [81].

HEOs with a combination of fully reactive elements have different reaction potentials for individual elements, and thus typically exhibit a stepped lithium storage mechanism that can effectively mitigate volume expansion during cycling [106,107,108]. Exploring the mechanism of the conversion reaction of the elements is of great significance for improving the electrochemical performance of HEOs. Huang et al. investigated the lithiation/delithiation mechanism of spinel-structured HEOs at an atomic scale. The valence changes of the elements in CrMnFeCoNi)_3_O_4_ during charging and discharging were analyzed using EELS spectroscopy [109]. Figure 9a illustrates the EELS edge spectra of the elements Cr, Mn, Fe, Co, and Ni during charge/discharge processes, where the intensity ratio of the white line between L_3_ and L_2_ is defined as the valence state of the element, while the peak shift represents oxidation/reduction processes. From the initial moment to the discharge to 0.5 V, the peaks of the Cr and Mn elements move to lower energy levels, indicating that Cr^3+^ is reduced to Cr^2+^, while Mn^2+/3+^ is reduced to Mn^0^. However, the peak positions of the elements Fe, Co, and Ni do not change, suggesting that the reduction of these three elements does not occur during this process. When discharged to 0.01 V, Cr^2+^ is further reduced to Cr^0/2+^. Fe^+2/+3^, Co^+2/+3^, and Ni^2+^ are reduced to Fe^0/+2/+3^, Co^0/+2/+3^, and Ni^0/+2^, respectively. During recharging, the five elements are reduced to their initial states, but Cr can only be reduced to Cr^2+^, which is related to the formation of lithiation products. Detailed elemental valence changes are shown in Figure 9b.

Ex situ technology often provides false results due to exposure to the air and moisture, especially for highly reactive, highly lithiated electrodes [109,110,111]. Luo et al. [81] systematically investigated the charging and discharging mechanisms of cobalt-free (CrMnFeNiCu)_3_O_4_ using operando quick-scanning X-ray absorption spectroscopy and provided detailed capacity contributions to the conversion reactions occurring in the elements. Figure 9c shows the first-order derivative images of the XAS spectra of each element in the (CrMnFeNiCu)_3_O_4_ anode electrode during the first charge/discharge at the current density of 150 mA g^−1^. The energy transfer process of Mn is divided into two stages; the initial stage in which Mn^2+/3+^ is reduced to Mn^2+^ provides a charging capacity of about 250 mAh g^−1^ (≈2.0–0.6 V). In addition, Mn^2+^ is reduced to Mn^0^ at a capacity that varies in the range of 250–900 mAh g^−1^ (≈0.61–0.31 V). Cu provides a contribution to the initial capacity of 250 mAh g^−1^ between discharge and 0.61 V, as Cu^2+^ is reduced to Cu^0^. Cr does not undergo a reduction reaction until it is charged to 0.69 V. Then, Cr^3+^ is sequentially reduced to Cr^2+^ (≈0.44 V) and C^0^ (0.01 V). The capacity contribution of Fe occurs within 200–525 mAh g^−1^ (0.66–0.51 V) as well as 525–825 mAh g^−1^ (0.51–0.36 V), mainly through the sequential reduction of Fe^2+/+3^ to Fe^2+^ and Fe^0^. Ni^2+^ is reduced to Ni^0^ in the capacity range of 200–900 mAh g^−1^ (0.66–0.31 V). During charging, the element Mn is rapidly oxidized, while the element Cu is unoxidized, indicating that the reaction of Cu is irreversible. The elements Cr, Fe, and Ni all require high potentials for oxidation to occur. The detailed transition behavior of the constituent elements during charging and discharging is shown in Figure 9d. 

**Figure 10:**
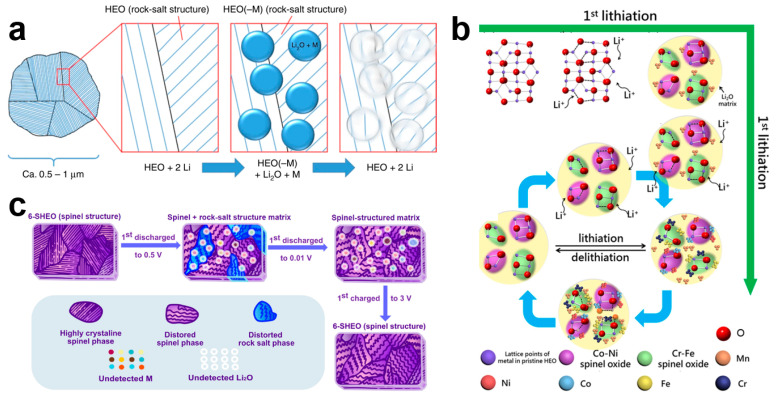
(**a**) Schematics of the proposed delithiation during the conversion reaction of (MgCoNiCuZn)O [38]. (**b**) Schematic illustration of the atomic-scale microstructure evolution of HEOs during lithiation/delithiation cycling [109]. (**c**) Illustration reflecting the lithium storage mechanism of the (FeNiCrMnMgAl)_3_O_4_ anode [98].

HEOs possess complex lithium storage mechanisms related to their diverse crystal structures and rich elemental combinations. A few representative lithium storage mechanisms are listed in this section: (1) In the (MgCoNiCuZn)O rock salt lattice as a substrate in the cycling process, the active elements provide capacity through redox reactions and the inactive elements act as structural stabilizers (Figure 10a) [38]. (2) Spinel (CrMnFeCoNi)_3_O_4_ HEOs are transformed into spinel Cr_x_Fe_3−x_O_4_ and LiNi_x_Co_1−x_O_2_ phases during lithiation, accompanied by Mn nanocrystals. These two spinel phases and Mn nanocrystals are embedded in a Li_2_O matrix, which acts as a buffer layer to mitigate the volume expansion during cycling (Figure 10b) [109]. (3) Highly crystalline long-range ordered spinel (FeNiCrMnMgAl)_3_O_4_ HEOs appear as short-range ordered rock salt and metal phases during discharge in a spinel matrix, completing the conversion reaction. During subsequent charging, the spinel structure is preserved but turns into chaotic spinel phases (Figure 10c) [97].

## 5. Summary and Outlook

In this review, we present a detailed overview of the preparation and crystal structure of and recent advances in the field of porous HEO anodes for lithium-ion batteries. As a new type of multi-component single-phase solid solution products, high-entropy oxides exhibit an excellent cycling stability and ultra-high room-temperature conductivity and are expected to break through the technical bottleneck of traditional transition metal oxide anodes. Although there have been many reports on HEOs, the development of HEO materials is still in its early stages, and there is still much room for improvement. For example, there is a lack of effective means to predict the synthesis of HEOs. If a method can be developed to accurately and quickly predict elemental combinations, it will be of vital importance for the design and expansion of applications of HEOs. In addition, conventional processes for the preparation of HEOs often require higher temperatures and longer times to promote the synthesis of single-phase solid solutions, which leads to the size transition growth of particles or even the emergence of dense microstructures, which seriously affect the application of HEOs. Therefore, it is of great value to develop a preparation process with a uniform distribution of the synthesized elements and a controllable microstructure.

In summary, in light of the current research status of porous HEOs in the field of energy storage, the following points are suggested to be considered for future studies.

(1) Design and development of new ingredients: Compositional design using high-throughput computing and machine learning and other means to assist high-entropy oxides exploitation, including compositional modulation in a wider range. At the same time, it is necessary to reduce the use of expensive elements as well as harmful elements.
(2) Development of new preparation methods: The low-energy, fast, and efficient preparation of HEOs requires the precise control of the synthesis conditions based on the two key factors of the desired size and morphology, as well as a high yield.
(3) Design of a new structure: Conventional nanoparticles tend to exhibit low initial coulombic efficiencies during electrochemical cycling, limiting the practical application of HEOs. The development of novel microstructures, such as biphasic high-entropy oxides, doped second phases, localized heterogeneous structures, etc., is expected to generate a new lithiation mechanism during charging and discharging, thus improving the lithium storage performance.
(4) Exploration of lithium storage mechanisms: HEOs with multiple active sites lead to complex lithium storage mechanisms, which are still subject to some controversy in the existing reports. Some advanced characterization techniques should be applied to reveal the truth and focus on the phase transitions of single-element effects and the synergistic effects among multiple elements.

There is no doubt that porous HEOs have been recognized as a new generation of anode materials for lithium-ion batteries with high capacity and stability. Although there is still considerable room for improvement, HEOs have the potential to be the anode materials for the next generation of batteries. In conclusion, we hope that this review will lay the foundation for the continued exploration of porous high-entropy oxides.

## Figures and Tables

**Table 1:** Pore structure characteristics of porous HEOs.

Composition	Preparation Method	Pore Size	Specific Surface Area (m^2^ g^−1^)	Ref.
(Mg_0.2_Ti_0.2_Zn_0.2_Cu_0.2_Fe_0.2_)_3_O_4_	Solid-state	3–20 nm	12.31	[35]
(FeCoNiCrMnCuLi)_3_O_4_	Solid-state	10–100 nm	1.674	[70]
(CrMnFeNiCu)_3_O_4_	Wet chemical	2–18 nm	13.66	[71]
(Al_0.2_CoCrFeMnNi)_0.58_O_4−δ_	Wet chemical	2–20 nm	44.86	[54]
(La_0.2_Y_0.2_Gd_0.2_Ce_0.2_Sm_0.2_)Zr_2_O_7_	Wet chemical	2–100 μm	-	[72]
(Ce_0.2_Zr_0.2_Ti_0.2_Sn_0.2_Ga_0.2_)O_2−δ_	Wet chemical	20–30 μm	-	[73]
(Co_0.2_Cu_0.2_Mg_0.2_Ni_0.2_Zn_0.2_)O	Wet chemical	10–145 nm	43.54	[68]
La(Co_0.2_Al_0.2_Fe_0.2_Mn_0.2_Cu_0.2_)O_3_	Wet chemical	6–20 nm	20.71	[74]

**Table 2:** Comparison and electrochemical performance of HEO anodes.

Composition	Structure	Method	Current Density (mA g^−1^)	Cycle Number	Reversible Capacity (mAh g^−1^)	Ref.
(Co_0.2_Cu_0.2_Mg_0.2_Ni_0.2_Zn_0.2_)O	Rock salt	NSP	200	500	590	[38]
Mg_0.2_Co_0.2_Ni_0.2_Cu_0.2_Zn_0.2_O	Rock salt	SRM	100	300	920	[57]
(MgCoNiCuZn)O	Rock salt	SRM	100	300	920	[90]
(MgCoNiCuZn)O	Rock salt	SCS	C/5	100	350	[91]
(MgCoNiCuZn)O	Rock salt	NSP	200	600	330	[92]
(MgCoNiCuZn)O	Rock salt	SRM	120	200	350	[93]
(MgCoNiCuZnLi)O	Rock salt	Molten salt	1000	100	610	[94]
(Mg_0.2_Ti_0.2_Zn_0.2_Cu_0.2_Fe_0.2_)_3_O_4_	Spinel	SRM	2000	800	272	[35]
(FeCoNiCrMn)_3_O_4_	Spinel	SRM	500	300	402	[21]
(CoCrFeMnNi)_3_O_4_	Spinel	SCS	2000	200	500	[95]
(FeCoNiCrMn)_3_O_4_	Spinel	SRM	2000	1200	596.5	[96]
(Cr_0.2_Mn_0.2_Fe_0.2_Co_0.2_Ni_0.2_)_3_O_4_	Spinel	SRM	2000	3000	244	[97]
(FeNiCrMnMgAl)_3_O_4_	Spinel	SCS	200	200	657	[98]
(Co_0.2_Cr_0.2_Fe_0.2_Mn_0.2_Ni_0.2_)_3_O_4_	Spinel	SCS	100	50	980	[87]
(FeCoNiCrMnCuLi)_3_O_4_	Spinel	SRM	500	100	522.1	[70]
(CrNiMnFeCu)_3_O_4_	Spinel	SCS	500	400	685	[99]
(Co_0.2_Ni_0.2_Mn_0.2_Zn_0.2_Fe_0.2_)_3_O_3.2_	Spinel	SCS	100	200	625	[100]
[(NaBi)_0.2_(LiLa)_0.2_(CeK)_0.2_Ca_0.2_Sr_0.2_]TiO_3_	Perovskite	SRM	100	50	85	[101]

## Data Availability

Not applicable.

## References

[1] Lee M.J., Han J., Lee K., Lee Y.J., Kim B.G., Jung K.N., Kim B.J., Lee S.W. (2022). Elastomeric electrolytes for high-energy solid-state lithium batteries. Nature.

[2] Aini Q., Irmawati Y., Karunawan J., Pasha M.H.R., Alini A., Iskandar F., Sumboja A. (2023). Para grass-derived porous carbon-rich SiO_x_/C as a stable anode for lithium-ion batteries. Nature.

[3] Farahmandjou M., Zhao S., Lai W., Sun B., Notten P., Wang G. (2022). Oxygen redox chemistry in lithium-rich cathode materials for Li-ion batteries: Understanding from atomic structure to nano-engineering. Nano Mater. Sci..

[4] Dubarry M., Costa N., Matthews D. (2023). Data-driven direct diagnosis of Li-ion batteries connected to photovoltaics. Nat. Commun..

[5] Nguyen Thi X.M., Le K.M., Phung Q., Truong D.Q., Nguyen H.V., Nguyen Q.N., Huynh T.T.K., Pham L.T., Van M.T., Le P.M.L. (2023). Improving the electrochemical performance of lithium-ion battery using silica/carbon anode through prelithiation techniques. Battery Energy.

[6] Wang Z.F., Yan Y.J., Zhang Y.G., Chen Y.X., Peng X.Y., Wang X., Zhao W.M., Qin C.L., Liu Q., Liu X.J. (2023). Single-atomic Co-B_2_N_2_ sites anchored on carbon nanotube arrays promote lithium polysulfide conversion in lithium–sulfur batteries. Carbon Energy.

[7] Wang Z., Wang H., Liu X., Chen Y., Zhao Y., Zhang Y., Han Q., Qin C., Bakenov Z., Wang Y. (2023). Single Zn atoms anchored on hollow carbon nanofiber network for dendrite-free lithium metal anode of flexible Li–S full cell. Rare Met..

[8] Lee B.S., Oh S.H., Choi Y.J., Yi M.J., Kim S.H., Kim S.Y., Sung Y.E., Shin S.Y., Lee Y.J., Yu S.H. (2023). SiO-induced thermal instability and interplay between graphite and SiO in graphite/SiO composite anode. Nat. Commun..

[9] Ge M.Z., Cao C.Y., Biesold G.M., Sewell C.D., Hao S.M., Huang J.Y., Zhang W., Lai Y.K., Lin Z.Q. (2021). Recent advance in silicon-based electrodes: From fundamental research toward practical applications. Adv. Mater..

[10] Kim H.S., Kim D.W., Kim S.S., Senthil C., Jung H.Y. (2022). Freestanding conversion-type anode via one-pot formation for flexible Li-ion battery. Chem. Eng. J..

[11] Ma Y.J., Ma Y., Wang Q.S., Schweidler S., Botros M., Fu T.T., Hahn H., Brezesinski T., Breitung B. (2021). High-entropy energy materials: Challenges and new opportunities. Energy Environ. Sci..

[12] Liu X.F., Li X.K., Zhang H.J., Jia Q.L., Zhang S.W., Lei W. (2022). High-entropy oxide: A future anode contender for lithium-ion battery. EcoMat..

[13] Salian A., Mandal S. (2021). Entropy stabilized multicomponent oxides with diverse functionality—A review. Crit. Rev. Solid State Mater. Sci..

[14] Lin Y., Luo N., Chamas M., Hu C.F., Grasso S. (2021). Sustainable high-entropy ceramics for reversible energy storage: A short review. Int. J. Appl. Ceram. Technol..

[15] Sun Z., Zhao Y.J., Sun C., Ni Q., Wang C.Z., Jin H.B. (2022). High entropy spinel-structure oxide for electrochemical application. Chem. Eng. J..

[16] Murthy A.A., Subiantoro A., Norris S., Fukuta M. (2019). A review on expanders and their performance in vapour compression refrigeration systems. Int. J. Refrig..

[17] Chen K.P., Pei X.T., Tang L., Cheng H.R., Li Z.M., Li C.W., Zhang X.W., An L.N. (2018). A five-component entropy-stabilized fluorite oxide. J. Eur. Ceram. Soc..

[18] Sarkar A., Djenadic R., Wang D., Hein C., Kautenburger R., Clemens O., Hahn H. (2018). Rare earth and transition metal based entropy stabilised perovskite type oxides. J. Eur. Ceram. Soc..

[19] Rost C.M., Sachet E., Borman T., Moballegh A., Dickey E.C., Hou D., Jones J.L., Curtarolo S., Maria J.P. (2015). Entropy-stabilized oxides. Nat. Commun..

[20] Bérardan D., Franger S., Dragoe D., Meena A.K., Dragoe N. (2016). Colossal dielectric constant in high entropy oxides. Phys. Status Solidi RRL.

[21] Liu Z., Yuan X., Zhang S., Wang J., Huang Q., Yu N., Zhu Y., Fu L., Wang F., Chen Y. (2019). Three-dimensional ordered porous electrode materials for electrochemical energy storage. NPG Asia Mater..

[22] Wang D., Jiang S.D., Duan C.Q., Mao J., Dong Y., Dong K.Z., Wang Z.Y., Luo S.H., Liu Y.G., Qi X.W. (2020). Spinel-structured high entropy oxide (FeCoNiCrMn)_3_O_4_ as anode towards superior lithium storage performance. J. Alloys Compd..

[23] Marques O.J.B.J., Walter M.D., Timofeeva E.V., Segre C.U., Kim D.E., Hong S.L. (2023). Effect of initial structure on performance of high-entropy oxide anodes for li-ion batteries. Batteries.

[24] Sarkar A., Djenadic R., Usharani N.J., Sanghvi K.P., Chakravadhanula V.S.K., Gandhi A.S., Hahn H., Bhattacharya S.S. (2017). Nanocrystalline multicomponent entropy stabilised transition metal oxides. J. Eur. Ceram. Soc..

[25] Triolo C., Maisuradze M., Li M., Liu Y.C., Ponti A., Pagot G., Di Noto V., Aquilanti G., Pinna N., Giorgetti M. (2023). Charge storage mechanism in electrospun spinel-structured high-entropy (Mn_0.2_Fe_0.2_Co_0.2_Ni_0.2_Zn_0.23_O_4_ oxide nanofibers as anode material for li-ion batteries. Small.

[26] Minouei H., Tsvetkov N., Kheradmandfard M., Han J.H., Kim D.E., Hong S.L. (2022). Tuning the electrochemical performance of high-entropy oxide nanopowder for anode Li-ion storage via structural tailoring. J. Power Sources.

[27] Shin D., Chae S., Park S., Seo S., Choi W. (2023). Rational engineering of high-entropy oxides for Li-ion battery anodes with finely tuned combustion syntheses. NPG Asia Mater..

[28] Bayraktar D.O., Lokcu E., Ozgur C., Erdil T., Toparli C. (2022). Effect of synthesis environment on the electrochemical properties of (FeMnCrCoZn)_3_O_4_ high-entropy oxides for li-ion batteries. Int. J. Energy Res..

[29] Suryanarayana C. (2019). Mechanical alloying: A novel technique to synthesize advanced materials. Research.

[30] Tallarita G., Licheri R., Garroni S., Orrù R., Cao G. (2019). Novel processing route for the fabrication of bulk high-entropy metal diborides. Scr. Mater..

[31] Meisenheimer P.B., Kratofil T.J., Heron J.T. (2017). Giant enhancement of exchange coupling in entropy-stabilized oxide heterostructures. Sci. Rep..

[32] Yang Z.M., Zhang K., Qiu N., Zhang H.B., Wang Y., Chen J. (2019). Effects of helium implantation on mechanical properties of (Al_0.31_Cr_0.20_Fe_0.14_Ni_0.35_)O high entropy oxide films. Chin. Phys. B.

[33] Bunpang K.K., Singkammo S., Cann D.P., Raengthon N. (2024). Titanate-based high-entropy perovskite oxides relaxor ferroelectrics. Sci. Rep..

[34] Dong Q., Hong M., Gao J.L., Li T.Y., Cui M.J., Li S.K., Qiao H.Y., Brozena A.H., Yao Y.G., Wang X.Z. (2022). Rapid synthesis of high-entropy oxide microparticles. Small..

[35] Chen H., Qiu N., Wu B.Z., Yang Z.M., Sun S., Wang Y. (2020). A new spinel high-entropy oxide (Mg_0.2_Ti_0.2_Zn_0.2_Cu_0.2_Fe_0.2_)_3_O_4_ with fast reaction kinetics and excellent stability as an anode material for lithium ion batteries. RSC Adv..

[36] Chellali M.R., Sarkar A., Nandam S.H., Bhattacharya S.S., Breitung B., Hahn H., Velasco L. (2019). On the homogeneity of high entropy oxides: An investigation at the atomic scale. Scr. Mater..

[37] Kamecki B., Karczewski J., Cempura G., Jasinski P., Molin S. (2023). Evaluation of structural and electrical properties of multicomponent spinel oxide thin films deposited via spray pyrolysis technique. Mater. Charact..

[38] Sarkar A., Velasco L., Wang D., Wang Q., Talasila G., Biasi L., Kübel C., Brezesinski T., Bhattacharya S.S., Hahn H. (2018). High entropy oxides for reversible energy storage. Nat. Commun..

[39] Goncalves J.M., Ghorbani A., Ritter T.G., Lima I.S., Saray M.T., Phakatkar A.H., Silva V.D., Pereira R.S., Yarin A.L., Angnes L. (2023). Multimetallic glycerolate as a precursor template of spherical porous high-entropy oxide microparticles. J. Colloid Interface Sci..

[40] Salian A., Pujar P., Vardhan R.V., Cho H.W., Kim S., Mandal S. (2023). Evolution of high dielectric permittivity in low-temperature solution combustion-processed phase-pure high entropy oxide (CoMnNiFeCr)O for thin film transistors. J. Eur. Ceram. Soc..

[41] He L., Kang H.J., Hou G.Y., Qiao X.S., Jia J., Qin W., Wu X.H. (2023). Low-temperature synthesis of nano-porous high entropy spinel oxides with high grain boundary density for oxygen evolution reaction. Chem. Eng. J..

[42] Nikiforova G.E., Kondrat’eva O.N., Tyurin A.V., Ryumin M.A., Guskov V.N., Gavrichev K.S. (2019). Thermophysical properties of M′-LuTaO_4_: Structural and calorimetric studies. J. Alloys Compd..

[43] Spiridigliozzi L., Dell’Agli G., Callone E., Dirè S., Campostrini R., Bettotti P., Bortolotti M., Speranza G., Sglavo V.M., Biesuz M. (2022). A structural and thermal investigation of Li-doped high entropy (Mg,Co,Ni, Cu, Zn)O obtained by co-precipitation. J. Alloys Compd..

[44] Kotsonis G.N., Rost C.M., Harris D.T., Maria J.P. (2018). Epitaxial entropy-stabilized oxides: Growth of chemically diverse phases via kinetic bombardment. MRS Commun..

[45] Sharma Y., Musico B.L., Gao X., Hua C., May A.F., Herklotz A., Rastogi A., Mandrus D., Yan J., Lee H.N. (2018). Single-crystal high entropy perovskite oxide epitaxial films. Phys. Rev. Mater..

[46] Tomboc G.M., Zhang X., Choi S., Kim D., Lee L.Y.S., Lee K. (2022). Stabilization, characterization, and electrochemical applications of high-entropy oxides: Critical assessment of crystal phase–properties relationship. Adv. Funct. Mater..

[47] Biesuz M., Chen J., Bortolotti M., Speranza G., Esposito V., Sglavo V.M. (2022). Ni-free high-entropy rock salt oxides with Li superionic conductivity. J. Mater. Chem. A.

[48] Dąbrowa J., Stygar M., Mikuła A., Knapik A., Mroczka K., Tejchman W., Danielewski M., Martin M. (2018). Synthesis and microstructure of the (Co,Cr,Fe,Mn,Ni)_3_O_4_ high entropy oxide characterized by spinel structure. Mater. Lett..

[49] Ko S.T., Lee T., Qi J., Zhang D.W., Peng W.T., Wang X., Tsai W.C., Sun S.K., Wang Z.K., Bowman W.J. (2023). Compositionally complex perovskite oxides: Discovering a new class of solid electrolytes with interface-enabled conductivity improvements. Matter.

[50] Wright A.J., Wang Q.Y., Hu C.Z., Yeh Y.T., Chen R.K., Luo J. (2021). Single-phase duodenary high-entropy fluorite/pyrochlore oxides with an order-disorder transition. Acta Mater..

[51] Gild J., Samiee M., Braun J.L., Harrington T., Vega H., Hopkins P.E., Vecchio K., Luo J. (2018). High-entropy fluorite oxides. J. Eur. Ceram. Soc..

[52] Walczak K., Plewa A., Ghica C., Zajac W., Trenczek Z.A., Zajac M., Tobo J., Molenda J. (2022). NaMn_0.2_Fe_0.2_Co_0.2_Ni_0.2_Ti_0.2_O_2_ high-entropy layered oxide—Experimental and theoretical evidence of high electrochemical performance in sodium batteries. Energy Storage Mater..

[53] Kawaguchi T., Bian X., Hatakeyama T., Li H., Ichitsubo T. (2022). Influences of enhanced entropy in layered rock-salt oxide cathodes for lithium-ion batteries. ACS Appl. Energy Mater..

[54] Xiang H.Z., Xie H.X., Chen Y.X., Zhang H., Mao A.Q., Zheng C.H. (2021). Porous spinel-type (Al_0.2_CoCrFeMnNi)_0.58_O_4-δ_ high-entropy oxide as a novel high-performance anode material for lithium-ion batteries. J. Mater. Sci..

[55] Ponti A., Triolo C., Petrovicova B., Ferretti A.M., Pagot G., Xu W.L., Di N.V., Pinna N., Santangelo S. (2023). Structure and magnetism of electrospun porous high-entropy (Cr_1/5_Mn_1/5_Fe_1/5_Co_1/5_Ni_1/5_)_3_O_4_, (Cr_1/5_Mn_1/5_Fe_1/5_Co_1/5_Zn_1/5_)_3_O_4_ and (Cr_1/5_Mn_1/5_Fe_1/5_Ni_1/5_Zn_1/5_)_3_O_4_ spinel oxide nanofibers. Phys. Chem. Chem. Phys..

[56] Yang X.B., Wang H.Q., Song Y.Y., Liu K.T., Huang T.T., Wang X.Y., Zhang C.F., Li J. (2022). Low-temperature synthesis of a porous high-entropy transition-metal oxide as an anode for high-performance lithium-ion batteries. ACS Appl. Mater. Interfaces..

[57] Wang Q.S., Sarkar A., Li Z.Y., Lu Y., Velasco L., Bhattacharya S.S., Brezesinski T., Hahn H., Breitung B. (2019). High entropy oxides as anode material for Li-ion battery applications: A practical approach. Electrochem. Commun..

[58] Djenadic R., Sarkar A., Clemens O., Loho C., Botros M., Chakravadhanula V.S.K., Kübel C., Bhattacharya S.S., Gandhi A.S., Hahn H. (2016). Multicomponent equiatomic rare earth oxides. Mater. Res. Lett..

[59] Jiang S.C., Hu T., Gild J., Zhou N.X., Nie J.Y., Qin M., Harrington T. (2018). A new class of high-entropy perovskite oxides. Scr. Mater..

[60] Wang J.B., Cui Y.Y., Wang Q.S., Wang K., Huang X.H., Stenzel D., Sarkar A., Azmi R., Bergfeldt T., Bhattacharya S.S. (2020). Lithium containing layered high entropy oxide structures. Sci. Rep..

[61] Wang K., Hua W.B., Huang X.H., Stenzel D., Wang J.B., Ding Z.M., Cui Y.Y., Wang Q.S., Ehrenberg H., Breitung B. (2023). Synergy of cations in high entropy oxide lithium ion battery anode. Nat. Commun..

[62] Vinnik D.A., Trofimov E.A., Zhivulin V.E., Zaitseva O.V., Zherebtsov D.A., Starikov A.Y., Sherstyuk D.P., Gudkova S.A., Taskaev S.A. (2020). The new extremely substituted high entropy (Ba,Sr,Ca,La)Fe_6-x_(Al,Ti,Cr,Ga,In,Cu,W)_x_O_19_ microcrystals with magnetoplumbite structure. Ceram. Int..

[63] Dong Y., Ren K., Lu Y., Wang Q., Liu J., Wang Y. (2019). High-entropy environmental barrier coating for the ceramic matrix composites. J. Eur. Ceram. Soc..

[64] Kirnbauer A., Spadt C., Koller C.M., Kolozsvári S., Mayrhofer P.H. (2019). High-entropy oxide thin films based on Al–Cr–Nb–Ta–Ti. Vacuum.

[65] Feltrin A.C., Akhtar F. (2023). High-temperature oxidation kinetics of a metastable dual-phase diboride and a high-entropy diboride. J. Eur. Ceram. Soc..

[66] Tian L., Zhang Z., Liu S., Li G., Gao X. (2023). High-entropy perovskite oxide nanofibers as efficient bidirectional electrocatalyst of liquid-solid conversion processes in lithium-sulfur batteries. Nano Energy.

[67] Jothi P.R., Liyanage W., Jiang B., Paladugu S., Olds D., Gilbert D.A., Page K. (2022). Persistent structure and frustrated magnetism in high entropy rare-earth zirconates. Small.

[68] Yan X., Wang C., Ai T., Li Z., Niu Y. (2023). Synthesis of porous (Co_0.2_Cu_0.2_Mg_0.2_Ni_0.2_Zn_0.2_)O high entropy oxide catalysts for peroxymonosulfate activation toward tetracycline degradation. Inorg. Chem. Commun..

[69] Li H., Duan Y., Zhao Z., Cheng X., Bian W., Xiao Z. (2023). Atomic-scale storage mechanism in ultra-small size (FeCuCrMnNi)_3_O_4_/rGo with super-stable sodium storage and accelerated kinetics. Chem. Eng. J..

[70] Duan C.Q., Tian K.H., Li X.L., Wang D., Sun H.Y., Zheng R.G., Wang Z.Y., Liu Y.G. (2021). New spinel high-entropy oxides (FeCoNiCrMnXLi)_3_O_4_ (X = Cu, Mg, Zn) as the anode material for lithium-ion batteries. Ceram. Int..

[71] Nguyen T.X., Tsai C.C., Patra J., Clemens O., Chang J.K., Ting J.M. (2022). Co-free high entropy spinel oxide anode with controlled morphology and crystallinity for outstanding charge/discharge performance in lithium-ion batteries. Chem. Eng. J..

[72] An Y., Wan K., Song M., Zhao L. (2024). Thermal insulating and mechanical properties of high entropy pyrochlore oxide ceramic with hierarchical porous structures. Ceram. Int..

[73] Chen G., Li C., Li H., Wang L., Chen K., An L. (2021). Porous (Ce_0.2_Zr_0.2_Ti_0.2_Sn_0.2_Ga_0.2_)O_2–δ_ high-entropy ceramics with both high strength and low thermal conductivity. J. Eur. Ceram. Soc..

[74] Han B., Pan Q., Chen Y., Liu D., Zhou C., Xia K., Gao Q. (2023). High-entropy perovkite oxide washcoated porous alumina ceramic as a superb catalyst for activating peroxymonosulfate to eliminate refractory organic pollutants. Chem. Eng. J..

[75] Marques O.J., Chen C., Timofeeva E.V., Segre C.U. (2023). Local structure and conversion chemistry of high-entropy oxides as Li-ion anodes. J. Power Source.

[76] Dong L.S., Wang Z.G., Mi C., Zhao W.M., Qin C.L., Luo C., Wang Z.F. (2024). Defect-rich hierarchical porous spinel MFe_2_O_4_ (M = Ni, Co, Fe, Mn) as high-performance anode for lithium ion batteries. Mater. Today Chem..

[77] Dong L.S., Wang Z.G., Li Y.Y., Jin C.H., Dong F.B., Zhao W.M., Qin C.L., Wang Z.F. (2023). Spinel-structured, multi-component transition metal oxide (Ni,Co,Mn)Fe_2_O_4−x_ as long-life lithium-ion battery anode material. Batteries.

[78] Wang Q., Sarkar A., Wang D., Velasco L., Azmi R., Bhattacharya S.S., Bergfeldt T., Düvel A., Heitjans P., Brezesinski T. (2019). Multi-anionic and-cationic compounds: New high entropy materials for advanced Li-ion batteries. Energy Environ. Sci..

[79] Moździerz M., Dąbrowa J., Stępień A., Zajusz M., Stygar M., Zając W., Danielewski M., Świerczek K. (2021). Mixed ionic-electronic transport in the high-entropy (Co,Cu,Mg,Ni,Zn)_1−x_Li_x_O oxides. Acta Mater..

[80] Xiao B., Wu G., Wang T.D., Wei Z.G., Sui Y.W., Shen B.L., Qi J.Q., Wei F.X., Zheng J.C. (2022). High-entropy oxides as advanced anode materials for long-life lithium-ion batteries. Nano Energy.

[81] Luo X.F., Patra J., Chuang W.T., Nguyen T.X., Ting J.M., Li J., Pao C.W., Chang J.K. (2022). Charge-discharge mechanism of high-entropy Co-free spinel oxide toward Li^+^ storge examined using operando quick-scanning X-ray absorption spectroscopy. Adv. Sci..

[82] Shin H., Lee Y.K., Lu W. (2023). Structural degradation of graphite anode induced by dissolved manganese ions in lithium-ion batteries. J. Power Sources.

[83] Ryu K., Lee M.J., Lee K.B., Lee S.W. (2022). ZnO-embedded expanded graphite composite anodes with controlled charge storage mechanism enabling operation of lithium-ion batteries at ultra-low temperatures. Energy Environ. Mater..

[84] Luo C., Wang Z.G., Chen Y.X., Zhao Y.M., Han Q.Q., Qin C.L., Wang Z.F. (2022). Eutectic-derived bimodal porous Ni@NiO nanowire networks for high-performance Li-ion battery anodes. Int. J. Energy Res..

[85] Wang Z.F., Zhang X.M., Liu X.L., Zhang W.Q., Zhang Y.G., Li Y.Y., Qin C.L., Zhao W.M., Bakenov Z. (2020). Dual-network nanoporous NiFe_2_O_4/_NiO composites for high performance Li-ion battery anodes. Chem. Eng. J..

[86] Wang Z.F., Zhang X.M., Liu X.L., Wang Y.C., Zhang Y.G., Li Y.Y., Zhao W.M., Qin C.L., Mukanova A., Bakenov Z. (2020). Bimodal nanoporous NiO@Ni-Si network prepared by dealloying method for stable Li-ion storage. J. Power Sources.

[87] Zhao J., Yang X., Huang Y., Du F., Zeng Y. (2021). Entropy stabilization effect and oxygen vacancies enabling spinel oxide highly reversible lithium-ion storage. ACS Appl. Mater. Interfaces.

[88] Nguyen T.X., Patra J., Chang J.K., Ting J.M. (2020). High entropy spinel oxide nanoparticles for superior lithiation–delithiation performance. J. Mater. Chem. A.

[89] Mozdzierz M., Swierczek K., Dabrowa J., Gajewska M., Hanc A., Feng Z.H., Cieslak J., Kadziolka G.M., Plotek J., Marzec M. (2022). High-Entropy Sn_0.8_(Co_0.2_Mg_0.2_Mn_0.2_Ni_0.2_Zn_0.2_)_2.2_O_4_ conversion- alloying anode material for Li-ion cells: Altered lithium storage mechanism, activation of Mg, and origins of the improved cycling stability. ACS Appl. Mater. Interfaces.

[90] Qiu N., Chen H., Yang Z.M., Sun S., Wang Y., Cui Y.H. (2019). A high entropy oxide (Mg_0.2_Co_0.2_Ni_0.2_Cu_0.2_Zn_0.2_O) with superior lithium storage performance. J. Alloys Compd..

[91] Cai Z.P., Ma C., Kong X.Y., Wu X.Y., Wang K.X., Chen J.S. (2022). High-performance PEO-based all-solid-state battery achieved by Li-conducting high entropy oxides. ACS Appl. Mater. Interfaces.

[92] Biesuz M., Spiridigliozzi L., Dell’Agli G., Bortolotti M., Sglavo V.M. (2018). Synthesis and sintering of (Mg, Co, Ni, Cu, Zn)O entropy-stabilized oxides obtained by wet chemical methods. J. Mater. Sci..

[93] Breitun B., Wang Q.S., Schiele A., Tripkovic D., Sarkar A., Velasco L., Wang D., Bhattacharya S.S., Hahn H., Brezesinski T. (2020). Gassing behavior of high-entropy oxide anode and oxyfluoride cathode probed using differential electrochemical mass spectrometry. Batter. Supercaps.

[94] Wei J.L., Rong K., Li X.L., Wang Y.C., Qiao Z.A., Fang Y.X., Dong X.J. (2022). Deep eutectic solvent assisted facile synthesis of low-dimensional hierarchical porous high-entropy oxides. Nano Res..

[95] Liu X.F., Xing Y.Y., Xu K., Zhang H.J., Gong M.X., Jia Q.L., Zhang S.W., Lei W. (2022). Kinetically accelerated lithium storage in high-Entropy (LiMgCoNiCuZn)O enabled by oxygen vacancies. Small.

[96] Grzesik Z., Smola G., Miszczak M., Stygar M., Dabrowa J., Zajusz M., Swierczek K., Danielewski M. (2019). Defect structure and transport properties of (Co,Cr,Fe,Mn,Ni)_3_O_4_ spinelstructured high entropy oxide. J. Eur. Ceram. Soc..

[97] Wang B., Yao J., Wang J.H., Chang A. (2022). Spinel-type high-entropy (Co_0.2_Mn_0.2_Fe_0.2_Zn_0.2_Ti_0.2_)_3_O_4_ oxides constructed from disordered cations and oxygen vacancies. J. Alloys Compd..

[98] Zheng Y., Wu X., Lan X.X., Hu R. (2021). A Spinel (FeNiCrMnMgAl)_3_O_4_ high entropy oxide as a cycling stable anode material for Li-ion batteries. Processes.

[99] Patra J., Nguyen T.X., Tsai C.C., Clemens O., Li J., Pal P., Chan W.K., Lee C.H., Chen H.Y.T., Ting J.M. (2022). Effects of elemental modulation on phase purity and electrochemical properties of Co-free high-entropy spinel oxide anodes for lithium-ion batteries. Adv. Funct. Mater..

[100] Su L., Ren J.K., Lu T., Chen K.X., Ouyang J.W., Zhang Y., Zhu X.Y., Wang L.Y., Min H.H., Luo W. (2023). Deciphering structural origins of highly reversible lithium storage in high entropy oxides with in situ transmission electron microscopy. Adv. Mater.

[101] Nowak M., Walczak K., Milewska A., Plotek J., Budziak J., Molenda J. (2023). Electrochemical performance of different high-entropy cathode materials for Na-ion batteries. J. Alloys Compd..

[102] Beere H.K., Saray N.S., Reddy X.Z., Kulkarni P., Samanta K., Jung H.Y., Ghosh D. (2024). Compositionally complex ball-in-ball type metal oxide anode via laser-induced fast fabrication for binder-free high-capacity Li-ion batteries. ACS Nano.

[103] Bano A., Noked M., Major D.T. (2023). Theoretical insights into high-entropy Ni-Rich layered oxide cathodes for low-strain li-ion batteries. Chem. Mater..

[104] Brandt T.G., Tuokkola A.R., Yu M.J., Laine R.M. (2023). Liquid-feed flame spray pyrolysis enabled synthesis of Co- and Cr-free, high-entropy spinel oxides as Li-ion anodes. Chem. Eng. J..

[105] Chen T.Y., Wang S.Y., Kuo C.H., Huang S.C., Lin M.H., Li C.H., Chen H.Y.T., Wang C.C., Liao Y.F., Lin C.C. (2020). In operando synchrotron X-ray studies of a novel spinel (Ni_0.2_Co_0.2_Mn_0.2_Fe_0.2_Ti_0.2_)_3_O_4_ high-entropy oxide for energy storage applications. J. Mater. Chem. A.

[106] Lokcu E., Anik M. (2024). Investigating the structural and lithium storage properties of high-entropy oxides in the Mg-Co-Ni-Cu-Zn-O system. Scr. Mater..

[107] Ritter T.G., Goncalves J.M., Stoyanov S., Ghorbani A., Shokuhfar T., Shahbazian Y.R. (2023). AlTiMgLiO medium entropy oxide additive for PEO-based solid polymer electrolytes in lithium ion batteries. J. Energy Storage.

[108] Nguyen T.X., Patra J., Tsai C.C., Xuan W.Y., Chen H.Y.T., Dyer M.S., Clemens O., Li J., Majumder S.B., Chang J.K. (2023). Secondary-phase-induced charge-discharge performance enhancement of Co-free high entropy spinel oxide electrodes for Li-ion batteries. Adv. Funct. Mater..

[109] Huang C.Y., Huang C.W., Wu M.C., Patra J., Nguyen T., Chang M.T., Clemens O., Ting J.M., Chang J.K., Wu W.W. (2021). Atomic-scale investigation of lithium/delithiation mechanism in high-entropy spinel oxide with superior electrochemical performance. Chem. Eng. J..

[110] Manojkumar S., Lang C.H., Wu S.H., Chang J.K., Rajan J. (2024). Systematic study of Co-free LiNi_0.9_Mn_0.07_Al_0.03_O_2_ Ni-rich cathode materials to realize high-energy density Li-ion batteries. J. Colloid Interface Sci..

[111] Kondo T., Matsumura K., Rozier P., Simon P., Machida K., Takeda S., Ishimoto S., Tamamitsu K., Iwama E., Naoi W. (2024). Enhancing the phase stability of γ-phase Li_3_VO_4_ for high-performance hybrid supercapacitors: Investigating influential factors and mechanistic insights. Chem. Mater..

